# VENTX induces expansion of primitive erythroid cells and contributes to the development of acute myeloid leukemia in mice

**DOI:** 10.18632/oncotarget.13563

**Published:** 2016-11-24

**Authors:** Eva Gentner, Naidu M. Vegi, Medhanie A. Mulaw, Tamoghna Mandal, Shiva Bamezai, Rainer Claus, Alpaslan Tasdogan, Leticia Quintanilla-Martinez, Alexander Grunenberg, Konstanze Döhner, Hartmut Döhner, Lars Bullinger, Torsten Haferlach, Christian Buske, Vijay P.S. Rawat, Michaela Feuring-Buske

**Affiliations:** ^1^ Institute of Experimental Cancer Research, CCC and University Hospital of Ulm, 89081 Ulm, Germanyxs; ^2^ Department of Internal Medicine I, University Hospital Freiburg, 79106 Freiburg, Germany; ^3^ Institute of Immunology, Ulm University, 89081 Ulm, Germany; ^4^ Institute of Pathology, University of Tübingen, 72076 Tübingen, Germany; ^5^ Department of Internal Medicine III, University Hospital Ulm, 89081 Ulm, Germany; ^6^ MLL Munich Leukemia Laboratory, 81377 Munich, Germany

**Keywords:** acute erythroleukemia, VENTX, AML1-ETO, homeobox gene, embryonic transcription factor

## Abstract

Homeobox genes are key regulators in normal and malignant hematopoiesis. The human Vent-like homeobox gene *VENTX*, a putative homolog of the *Xenopus laevis* Xvent-2 gene, was shown to be highly expressed in normal myeloid cells and in patients with acute myeloid leukemia. We now demonstrate that constitutive expression of VENTX suppresses expression of genes responsible for terminal erythroid differentiation in normal CD34^+^ stem and progenitor cells. Transplantation of bone marrow progenitor cells retrovirally engineered to express VENTX caused massive expansion of primitive erythroid cells and partly acute erythroleukemia in transplanted mice. The leukemogenic potential of VENTX was confirmed in the AML1-ETO transplantation model, as in contrast to AML1-ETO alone co-expression of AML1-ETO and VENTX induced acute myeloid leukemia, partly expressing erythroid markers, in all transplanted mice. VENTX was highly expressed in patients with primary human erythroleukemias and knockdown of VENTX in the erythroleukemic HEL cell line significantly blocked cell growth. In summary, these data indicate that VENTX is able to perturb erythroid differentiation and to contribute to myeloid leukemogenesis when co-expressed with appropriate AML oncogenes and point to its potential significance as a novel therapeutic target in AML.

## INTRODUCTION

It has been shown that early developmental genes play a major role in adult hematopoiesis including orchestrating self-renewal and differentiation of hematopoietic stem and progenitor cells. This is exemplified by the family of homeobox genes, known to be key regulators in embryogenesis, but also during normal postnatal hematopoiesis [1]. Furthermore, it was shown that the role of homeobox genes extends beyond normal hematopoiesis, being critically involved in leukemogenesis. This role is well established for clustered HOX genes such as HOXA9, but also for non-clustered homeobox genes such as MEIS1, which is one of the most potent co-factors for HOX driven leukemias, or the Parahox gene CDX2 [2–4]. We recently described the human Vent-like homeobox gene *VENTX*, a putative homolog of the *Xenopus laevis* Xvent-2 gene, as a novel regulatory hematopoietic gene, which in contrast to leukemogenic HOX genes such as HOXA9 and HOXA10 is highly expressed in normal myeloid cells, but not in early CD34^+^ stem and progenitor cells. Constitutive expression of *VENTX* in normal CD34^+^ human progenitor cells impaired lymphoid engraftment and fostered generation of myeloid cells, but failed to induce leukemia *in vivo*. This was in contrast to our observation that VENTX was highly expressed in human AML, in particular in patients with the translocation t(8;21) which is associated with the most frequent fusion gene AML1-ETO (AE) [5]. We now provide evidence that VENTX suppresses expression of erythroid master regulators, expands primitive erythroid cells and contributes to leukemogenesis in transplanted mice.

## RESULTS

### VENTX impairs expression of genes involved in erythroid differentiation and is highly expressed in patients with acute erythroid leukemia and polycythemia vera

**Figure 1:**
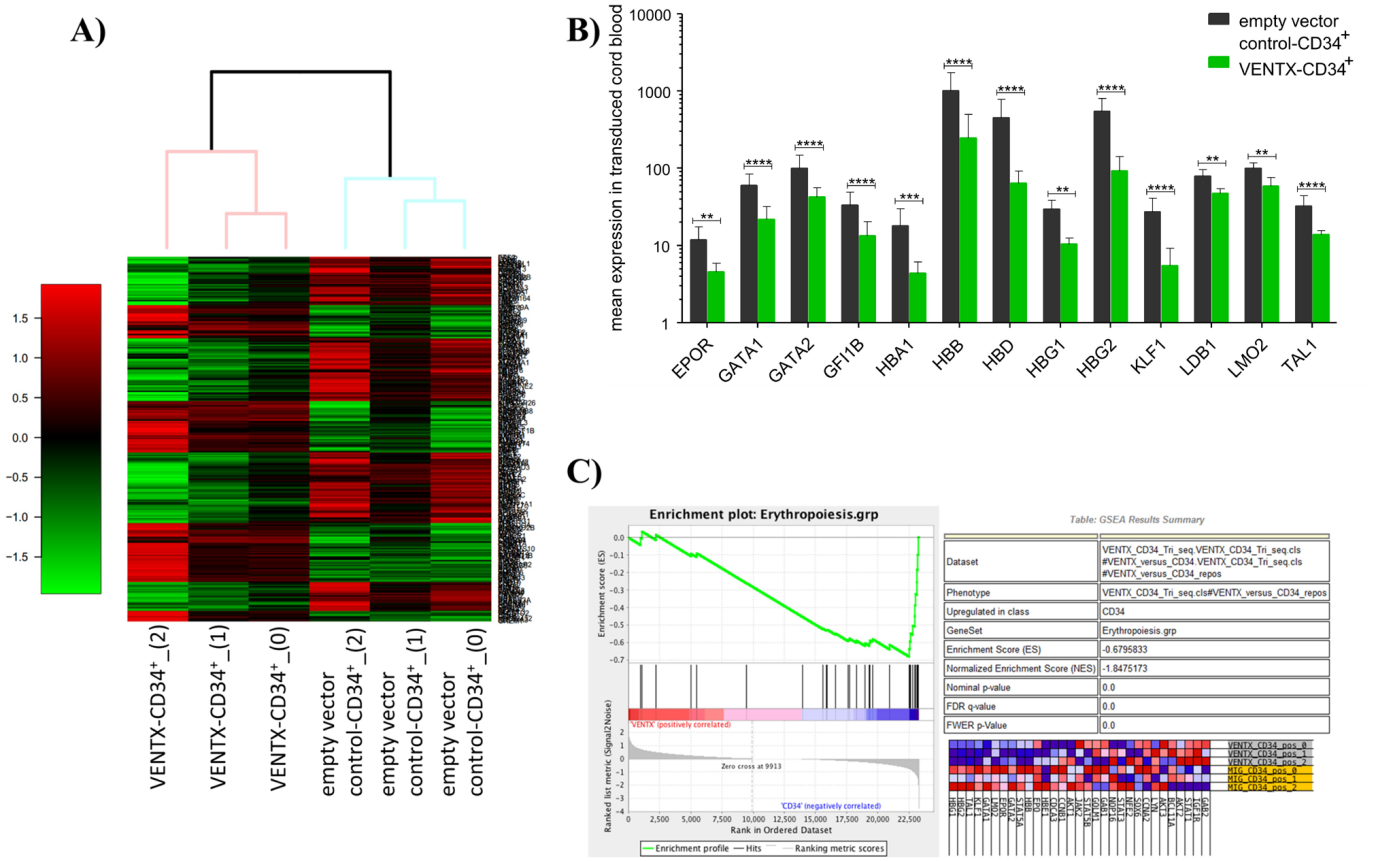
**A**. Heatmap of differentially expressed genes as determined by RNA-seq between CD34^+^ CB cells overexpressing VENTX compared to the empty vector control (n=3). **B**. Differentially expressed genes transcription factors, which are known to play a role in erythroid development. ****: p<0.0001, ***: p≤0.0001, **: p≤0.001. **C**. Gene set enrichment analysis of genes involved in erythropoietic differentiation.

Based on our previous findings that overexpression of VENTX is able to perturb normal hematopoietic differentiation, we first aimed at characterizing changes in the molecular profile induced by VENTX overexpression. For this VENTX was retrovirally expressed in normal human CD34^+^ cord blood (CB) cells for 48h before performing RNA-sequencing (RNA-Seq) (n=3). 278 genes were differentially expressed between VENTX and the empty vector control (Supplementary Table S1, Figure 1A). Pathway analyses showed changes in the expression of genes belonging to the categories JAK-STAT signaling pathway, Hematopoietic cell lineage, Cytokine-cytokine receptor interaction and Hemoglobin's Chaperone (Supplementary Figure S1A-S1C, Supplementary Table S2). Strikingly, overexpression of VENTX significantly downregulated genes involved in erythropoiesis, among them several genes known as master regulators of erythropoiesis: thus, genes such as GATA1 (2.7-fold, p<0.0001), GATA2 (2.3-fold, p<0.0001), LDB1 (1.7-fold, p=0.001), KLF1 (5.0-fold, p<0.0001), GFI1B (2.5-fold, p<0.0001), LMO2 (1.7-fold, p<0.001) and TAL1 (2.3-fold, p<0.0001) were downregulated in CD34^+^ CB cells constitutively expressing VENTX. In parallel genes involved in heme and hemoglobin synthesis were significantly downregulated such as HBA1 (4.1-fold, p=0.0001), HBB (4.1-fold, p<0.0001), HBD (7-fold, p<0.0001), HBG1 (2.8-fold, p<0.001), HBG2 (5.9-fold, p<0.0001) and EPOR (2.6-fold, p<0.001) (Figure 1B, Supplementary Table S1). Gene set enrichment analysis focusing on genes known to be involved in erythroid differentiation showed a highly significant negative enrichment in VENTX overexpressing cells compared to the empty vector control (Figure 1C). Of note, around every 10^th^ gene of our data set overlapped with proteins expressed during erythroid differentiation as recently shown by quantitative mass spectrometry to determine the absolute proteome composition of human erythroid progenitors throughout the differentiation process (Supplementary Table S3) [6].

**Figure 2:**
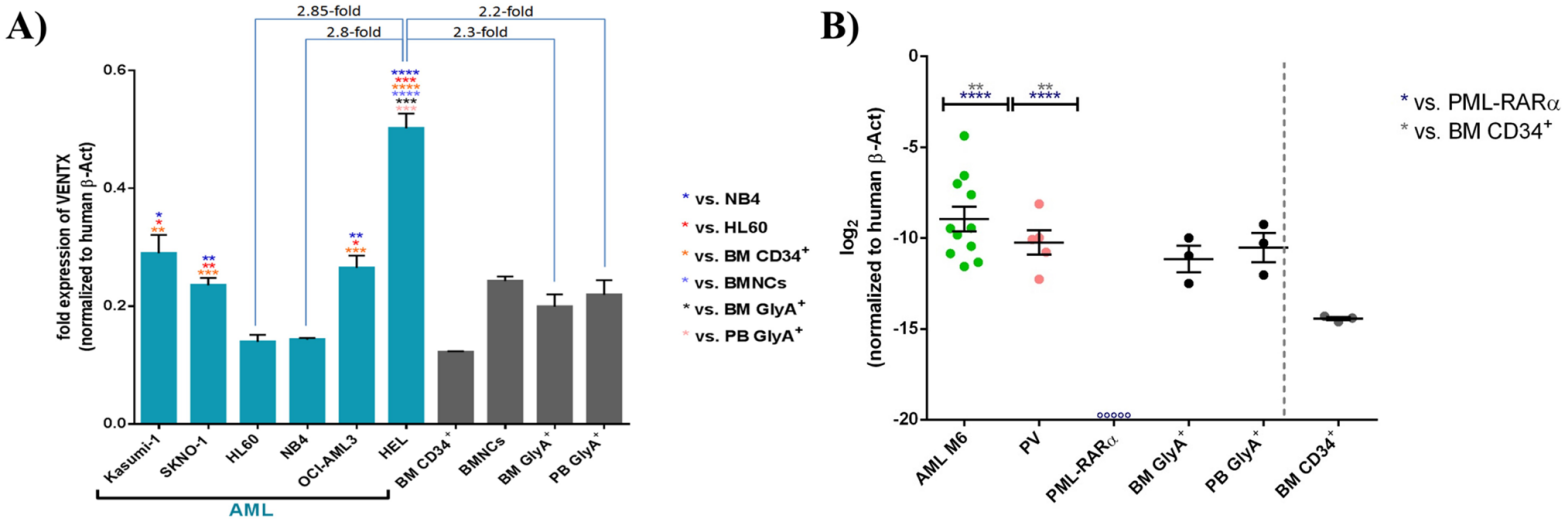
**A**. Quantitative expression of VENTX in different AML cell lines compared to BM CD34^+^/BMNCs/BM GlyA^+^/PB GlyA^+^. All expression analyses were performed by TaqMan^®^ qRT-PCR with (+)RT and (-)RT reaction samples. Fold expression values were obtained by normalizing the expression of the gene of interest (VENTX) to the endogenous human β-actin (β-Act). Bars are showing the average fold expression ± SEM. *: p≤0.05, **: p≤0.001, ***: p≤0.0001, ****: p<0.0001. **B**. Mean Quantitative expression of VENTX AML M6 and PML-RARα positive AML samples compared to Glycophorin A from BM/PB and CD34^+^ bone marrow cells. All expression analyses were performed by TaqMan^®^ qRT-PCR with (+)RT and (-)RT reaction samples. Log_2_ fold expression values were obtained by normalization of the expression of the gene of interest (VENTX) to the endogenous human β-actin. Bars are showing the log_2_ fold expression ± SEM. **: p≤0.001 and ****: p<0.0001. ○ indicates no detectable expression for 5 individual samples of PML-RARα positive AML cases (for up to 37 cycles).

As we saw suppression of genes driving erythroid maturation, we tested first the expression of VENTX in the human primitive erythroblastic cell line HEL compared to other myeloblastic cell lines and normal erythroid cells: of note, highest expression was seen in HEL with a significant and 2.8-fold higher expression than in HL60 cells (p<0.001). Furthermore, HEL expressed *VENTX* 2.2-fold and 2.3-fold higher than normal highly purified Glycophorin A positive peripheral blood or BM, respectively (p≤0.001) (Figure 2A). In line with data from primary AML samples [5], there was high expression in the t(8;21) positive cell lines Kasumi-1 and SKNO-1 (Figure 2A). Analyses were extended to primary erythroleukemias (AML M6) and polycythemia vera patient samples, documenting high expression of *VENTX* in clear contrast to PML-RARα positive AML, which did not show any detectable expression of VENTX as well as in contrast to CD34^+^ progenitor cells from normal bone marrow (Figure 2B, Table 1). To test whether expression levels of VENTX correlate with promoter methylation, DNA methylation of a total of 59 AML patients (54 normal karyotype (CN) AML samples, five samples with t(8;21)) was quantified by MassARRAY technology in comparison to normal CD34-enriched cord blood and PB of healthy individuals. A wide range of mean amplicon DNA methylation levels (4% to 86%) was observed in the set of 54 AML samples with normal karyotype for amplicon 1 versus low DNA methylation levels (10% to 21%) in hematopoietic progenitor cells from cord blood and in sorted subfractions from peripheral blood (10% to 19%). The t(8;21) positive AML samples did not cluster separately at the amplicon 1 region but exhibited overall low DNA methylation levels (7% to 20%) (Supplementary Figure S2A-S2C).

**Table 1:** Patients’ characteristics

Patient no.	Diagnosis	Gender	Age	Karyotype	Other relevant markers
1	AML M6a	M	77	46,XY,del(5)(q22q34),+8,dic(15;17)(p11;p11),der(20;21)(p10;q10),+der(20;21) (p10;q10) [16]	*DNMT3A, TP53*
2	AML M6a	M	48	46,XY,del(11)(p11p14) [11]46,XY [9]	*none*
3	AML M6	M	74	48,XY,+8,+8 [4]48,XY,der(6)t(6;6)(p25;q12),+8,+8 [15]46,XY [1]	*DNMT3A, RUNX1, TP53*
4	AML M6	F	81	46,XX,t(4;10)(q13;p12) [3]45,XX,t(4;10)(q13;p12),del(5)(q31q35),-7 [3]46,XX [4]	*TET2, TP53*
5	AML M6	M	71	47,XY,+8 [10]46,XY [10]	*ASXL1*
6	AML M6	F	38	46,XX	*ASXL1, FLT3-ITD, NPM1 mut*
7	AML M6: tAML	F	59	92-96,XXX,-X,2xadd(4)(q35),-7,+?12,+22,+1-3mar	*TET2*
8	AML M6: AML	M	56	46,XY,del(20)(q11) [5]46,XY [12]	*ASXL1, IDH2, MLL-PTD*
9	AML M6: sAML	F	57	48,XX,+8,+19	*TET2*
10	AML M6	F	42	46,XX,inv(9)(p11q13)c	*IDH1, NPM1 mut, WT1*
11	AML	M	75	45,X,-Y [16]46,XY [4]	*FLT3-ITD, NPM1 mut*
12	AML	F	45	46,XX [20]	*NPM1 mut*
13	AML	M	74	46,XY [20]	*FLT3-TKD, NPM1 mut*
14	AML	M	46	46,XY [20]	*FLT3-ITD, NPM1 mut*
15	AML	F	27	46,XX [21]	*NPM1 mut*
16	AML	F	46	46,XX,del(9)(q13q22) [10]46,XX [10]	*NPM1 mut*
17	AML	F	40	46,XX [21]	*FLT3-TKD, NPM1 mut*
18	AML	F	49	46,XX [23]	*FLT3-ITD, NPM1 mut*
19	PV	F	70	46,XX [18]	*JAK2*
20	PV	M	81	46,XY [20]	*DNMT3A,JAK2*
21	PV	M	60	46,XY [20]	*JAK2*
22	PV	F	65	46,XX [21]	*ASXL1,JAK2*
23	PV	M	76	46,XY [20]	*JAK2*

Expression levels of VENTX were compared to DNA methylation in 8 NPM1 mutated patient samples (Table 1) as well as in sorted CD3^+^, CD14^+^, CD15^+^, CD19^+^ and GlyA^+^ subfractions from PB and CD34^+^CD38^-^, CD34^+^CD38^+^, CD34^-^CD38^+^ subpopulations from CB, respectively. However, there was no significant correlation between the VENTX expression and the level of DNA methylation of amplicon 1 and amplicon 2 in all cell populations tested (data not shown).

**Figure 3:**
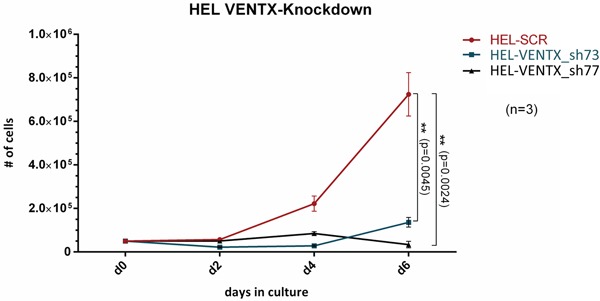
Cell proliferation in liquid expansion cultures from HEL cells after shRNA mediated knockdown of VENTX (shVENTX_73, shVENTX_77) compared to the scrambled control. Knockdown efficiency was 99% for both shRNA constructs (n=3).

To evaluate, whether leukemic cells depend on VENTX expression, the impact of shRNA mediated depletion of VENTX on growth of HEL cells was tested: knockdown of VENTX expression by 98.85% and 99.43% for shVENTX_73 and shVENTX_77, respectively, resulted in a mean reduction of cell growth of 81% (76%-84%) and 95% (91%-98%) after 6 days in liquid culture (n=3) (Figure 3). We extended these analyses to other leukemic cell lines such as K562 and OCI-AML3 cell lines. Similar findings were detected, when VENTX was knocked down in these cell lines with a significant reduction in cell proliferation, colony growth in methylcellulose, as well as survival and engraftment levels in NSG mice (for OCI-AML3) (data not shown). The efficiency of the shRNA mediated knockdown of VENTX on the protein level was measured after overexpression of VENTX in HEL cells by intracellular staining. As shown in Supplementary Figure S3 we could demonstrate, that knockdown of VENTX resulted in a decrease of VENTX protein as determined by a weaker fluorescent signal.

We performed an Annexin V staining and a BrdU assay to evaluate, whether cell cycle arrest, apoptosis or senescence might contribute to the oncogenic cooperativity in the AML1-ETO mouse model. Knockdown of VENTX lead to a 2.4-fold increase of the proportion of non-cycling cells as compared to the scrambled control arm. Using the Annexin V staining, we did not detect an increase of early or late apoptotic cells when VENTX was knocked down (data not shown).

### VENTX expands the primitive erythroid compartment and causes acute leukemia in transplanted mice

**Figure 4:**
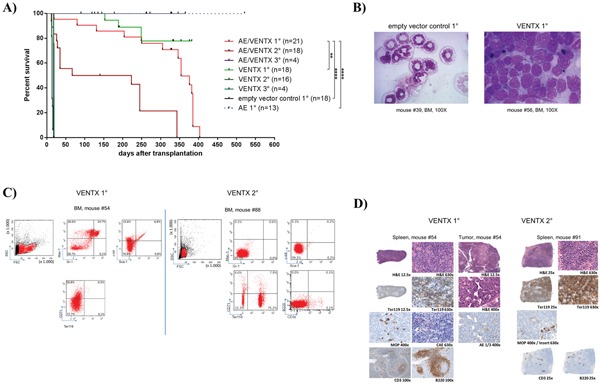
**A**. Kaplan-Meier survival curves of mice transplanted with 5-FU stimulated transduced BM cells expressing AML1-ETO+VENTX [AE/VENTX 1°] (n=20), VENTX [VENTX 1°] (n=18), empty vector control (n=18) and AML1-ETO [AE 1°] (n=15). Secondary and tertiary transplantations are shown for leukemic mice: AE/VENTX 2° (n=14), AE/VENTX 3° (n=9), VENTX 2° (n=19), VENTX 3° (n=4). AE/V 1° - V 1° V = VENTX: **, p= 0.0011; AE/V 1° - empty vector control 1°: ****, p<0.0001; AE/V 1° - AE1°: ****, p <0.0001; V 1° - empty vector control 1°: n.s. (n.s. = not significant); V 1° - AE1°: ns; AE/V 2° - V 2°: ns; AE/V 3° - V 3°: ns. **B**. Representative cytomorphological analyses of bone marrow of a diseased VENTX transplanted 1° mouse compared to an empty vector control mouse. **C**. Representative flow cytometric analysis of a primary VENTX recipient mouse (left side) and a secondary VENTX recipient mouse (right side) **D**. Histological analysis of primary (left side) and secondary (right side) VENTX recipient mice.

To extend analyses on the functional relevance of VENTX overexpression, the homeobox gene was constitutively expressed in mouse BM progenitor cells and transplanted into lethally irradiated mice. Of note, all transplanted mice (n=18) showed an accumulation of blasts in the BM and infiltration in the spleen with undifferentiated and erythroid blasts (median 47%, range 29%-85%). (Supplementary Table S4). 3 of 18 mice developed overt clinical disease and died after 153, 189 and 249 days, respectively (Figure 4A) with an infiltration of 29%, 85% and 60% blasts in the bone marrow, respectively. The blasts were mostly undifferentiated with a substantial proportion displaying an erythroblast phenotype (24%, 95% and 55%, respectively) (Figure 4B and Supplementary Table S4). Leukemias were readily transplantable in 13 of 19 mice tested with disease development after short latency (median 14 days, range 11-67 days) (Figure 4A) and high erythroblast counts in the bone marrow, spleen and peripheral blood in secondary mice (Supplementary Table S4, Figure 4B). These blasts lacked expression of myeloid markers and were highly positive for Ter119 (median 86.8%, range 76.9 – 95.2%) and partly CD71 (median 45%, range 1 – 79.3%) (Figure 4C and Supplementary Table S5). Histological analyses in VENTX diseased mice showed the presence of erythroblasts with the diagnosis of an erythroleukemia according to the Bethesda criteria for non-lymphoid hematopoietic neoplasms (Figure 4D) [7].

**Figure 5:**
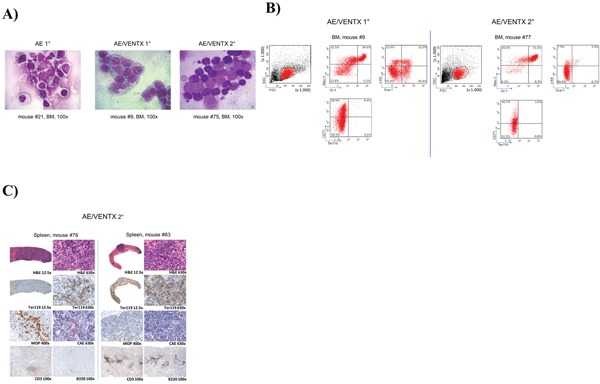
**A**. Representative cytomorphological analysis of bone marrow cytospins of diseased mice as indicated (AE/VENTX 1° and 2°) compared to a representative non-leukemogenic AE 1° mouse. **B**. Representative flow cytometric analysis of a primary AE/VENTX recipient mouse (left side) and a secondary AE/VENTX recipient mouse (right side) **C**. Histological analysis of two secondary transplanted AE/VENTX mice, showing erythroblasts in the spleen of the diseased animals.

**Figure 6:**
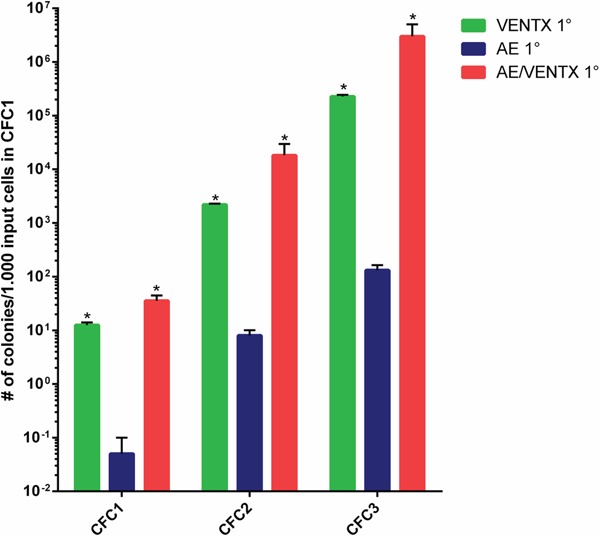
*Ex vivo* colony forming cell assay (CFC1) and re-plating assays (CFC2, CFC3) of leukemic cells isolated from mice transplanted initially with 5-FU BM cells transduced with the different constructs as indicated. Results are means ± SEM. The number of CFC refers to 1000 input cells in the primary CFC assay. For 2° and 3° CFC assay also the CFC numbers are indicated per initially plated 1000 cells for the primary CFC (*: p≤0.05).

To confirm the leukemogenic potential of VENTX we co-expressed the homeobox gene with the fusion gene AML1-ETO in the bone marrow transplantation assay. The rationale for this was that we have seen high expression of VENTX in patients with AML1-ETO positive leukemia among others before [5]. Furthermore, it was previously shown, that AE is only able to induce leukemia with a leukemogenic partner in transplanted mice [8–10]. All mice (n=20) transplanted with AE plus VENTX developed leukemia after a median of 337.5 days (range 19-403 days) post-transplantation (Figure 4A). Leukemias were characterized by high blast counts of mean 68.5% in the bone marrow (range 38 - 100%) (Supplementary Table S4, Figure 5A) and expression of progenitor cell and myeloid markers: Sca-1 (median 20.5%, range 11.8-32.4%) and c-kit (median 8.5%, range 2.9 – 78.5%), Gr-1 (median 34.7%, range 3.0 – 52.3%) and Mac-1 (median 33.2%, range 0-67.1%) (Figure 5B, Supplementary Table S5). Surprisingly, myeloid blasts expressed CD71 in the bone marrow, which we did not see in our AML1-ETO bone marrow transplantation model overexpressing FLT3-LM (Figure 5B) [8]. Strikingly, secondary diseased mice displayed partly large nodules consisting of erythroblasts extending to diffuse erythroblastic infiltration in the spleen in other mice. The white pulp was almost eradicated with few residual B220 and CD3 positive cells (Figure 5C). Leukemias were readily transplantable in secondary (n=14) and tertiary recipient mice (n=9) (median 129 days, range 13 to 343 days for secondary and median 14 days, range 13 to 18 days for tertiary mice) (Figure 4A). BM of diseased mice displayed an infiltration of the bone marrow with CD71 (median 46.7%, range 22.5 - 72%) and CD36 (median 82%, range 60.2 – 90.7%) expressing cells. To exclude retroviral insertional mutagenesis in VENTX and AE/VENTX induced leukemias, integration sites were sequenced: there was one, but not recurrent site enlisted in the retroviral tagged cancer genes database (RTCGD) (Supplementary Table S6). Leukemic cells originating from diseased AE/VENTX mice showed 250-fold (± 30) and 713-fold (± 90) increased colony formation and re-plating capacity *ex vivo*, respectively (p<0.05), compared to BM cells isolated from mice transplanted with cells expressing AE alone (Figure 6). Cytospins of generated colonies showed blast morphology with >50% of blast cells/blasts. Cells from a secondary AE/VENTX mouse showed permanent but growth factor (rmIL3, rmSCF, rmIL6) dependent cell growth, blast morphology, and high expression of CD71 (87.4%), whereas Ter119 as well as Gr-1, Mac-1, Sca-1 and c-kit were virtually not expressed, corresponding to the immunophenotype of erythroid precursors [11] (data not shown). This cell population maintained its leukemogenicity *in vivo* and rapidly induced leukemia in transplanted mice (n=3) within 14 days (13 - 14 days). Engrafted cells expressed Ter119 and maintained the erythroblastic phenotype (Supplementary Figure S4A-S4B). When placed in methylcellulose assay cells from this cell line generated undifferentiated serially replatable colonies, which expressed CD71 (85.6%) and were negative for Sca-1, c-kit, Ter119, Gr-1, Mac-1 (data not shown).

## DISCUSSION

This report documents for the first time that aberrant expression of the homeobox gene VENTX contributes to the development of acute myeloid leukemia and by this extends the list of non-clustered homeobox genes with leukemogenic potential. We reported previously that VENTX is highly expressed in various subtypes of AML, but failed to prove on a functional level that the gene is directly involved in myeloid leukemogenesis, possibly also because of the limitations of xenograft models to mirror the effect of oncogenes to a full extent [5]. In our current bone marrow transplantation model, aberrant expression of VENTX was able to induce acute leukemia in a part of transplanted mice after long latency, indicating that VENTX needs collaborative partners to exert its full leukemogenic potential. One of the candidates for such an oncogenic partner is the most frequent fusion gene in human AML, AML1-ETO, as we had seen high expression of VENTX in patients with AML1-ETO positive AML [5]. AML1-ETO is able to increase self-renewal of hematopoietic stem- and progenitor cells, but is not overt leukemogenic as shown in several independent mouse and human experimental models [8–10, 12–15]. VENTX induced AML in all transplanted mice in collaboration with AML1-ETO, characterizing VENTX as a novel collaborative partner of AML1-ETO. With this, VENTX is among the first homeobox genes shown to cooperate with AML1-ETO, as we could just recently demonstrate that the TALE homeobox gene MEIS2 collaborates with the fusion in inducing AML [16]. It also demonstrates that the transcription factor AML1-ETO can collaborate with its own class as VENTX is a bona fide transcription factor. Following the classical two hit model proposed previously [17, 18], leukemic transformation depends on two factors, one conferring self-renewal and the other one impairing differentiation of hematopoietic progenitor cells. AML1-ETO has a well-established function as a factor increasing self-renewal [19]. Although the two-hit model is surely an oversimplification of complex AML biology, it would require that VENTX impairs differentiation. Indeed, the most surprising observation was that VENTX induced expansion of primitive erythroid cells up to erythroblastic leukemia on its own and induced AML with partly erythroid features together with AML1-ETO. This went along with suppression of several erythroid master regulators of erythroid differentiation, resulting in a highly significant negative enrichment score for expression of these factors in VENTX overexpressing cord blood cells. We had not seen this erythroblastic phenotype in our own AML1-ETO - FLT3-LM model or from the perspective of homeobox genes in our AML models depending on homeobox gene expression [4, 8, 20, 21]. However, it has been described previously, that AML1-ETO positive leukemias show an impairment in erythroid differentiation [22–25]. This might indicate, that VENTX impairs in particular differentiation along the erythroid line in hematopoietic progenitor cells enforced to self-renew by the AML1-ETO fusion. In line with this overexpression of VENTX in CD34^+^ cord blood cells resulted in a nearly complete block of erythroid differentiation with an 81% reduction of the number of BFU-E as previously reported by us [5]. Our data demonstrate that aberrant expression of VENTX has leukemogenic potential. Importantly, Gao et al recently described that VENTX can act as a potential tumor suppressor gene in solid cancer cell lines originating from lung and colon cancer [26]. Furthermore, the same group previously demonstrated that VENTX is able to impair cell growth in chronic lymphocytic leukemia [27]. All these data indicate that the function of VENTX largely depends on the cellular context and that VENTX can act as tumor suppressor and as oncogene. This has to be taken into account, if therapeutic approaches are considered which are targeting VENTX dependent functions or VENTX itself.

Taken together, our data extend the number of homeobox genes critically involved in myeloid leukemogenesis, but also underline that the role of this gene family in tumorigenesis can critically depend on the cancer subtype.

## MATERIALS AND METHODS

### Human samples and cell lines

Mononuclear cells isolated from diagnostic bone marrow (BM) or peripheral blood (PB) from 28 adult patients were analyzed for their VENTX expression: n=10 for AML M6, n=5 for polycythaemia vera (PV) and n=5 for PML-RARα, n=8 for NPM1 mutated AML. CD34^+^ bone marrow mononuclear cells (BMNCs) and CD34^+^ cord blood (CB) cells (both from Lonza, Cologne, Germany) (n=5 and n=3) as well as sorted subfractions from peripheral blood and bone marrow from healthy individuals were taken as controls [5]. Cytomorphological, cytogenetic and molecular analyses were performed in all cases as described. Cases were classified according to the French-American-British criteria and the World Health Organization classification (Table 1) [28, 29]. The study was approved by the ethics committees of all participating institutions, and informed consent was obtained from all patients before they entered the study in accordance with the Declaration of Helsinki (http://www.wma.net/en/30publications/10policies/b3/index.html).

All cell lines used were obtained from DSMZ, Braunschweig, Germany. Original stocks were frozen down and all experiments were performed with aliquots from the original stock with less than 6 months propagation *in vitro*. DSMZ guarantees originality of cell lines by comprehensive molecular testing e.g. by polymerase chain reaction (PCR) amplification of eight highly polymorphic microsatellite STR loci, using the uniqueness of DNA profiles in an STR database. For RNA-Seq experiments, human cord blood cells were retrovirally transduced with VENTX or an empty vector control as described previously [5].

### Quantification of VENTX expression

Total RNA was isolated using the Direct-zol™ Kit (Zymo Research, Freiburg, Germany) and TRIzol^®^-Reagent. To avoid genomic DNA contamination all RNA samples were treated with *DNase*I enzyme according to the manufacturer's instructions (ThermoFisher, Darmstadt, Germany). cDNA was prepared using random hexamer primers (Primescript RT-PCR kit; TAKARA Clontech, Saint-Germain-en-Laye, France) according to the manufacturer's instructions. For endogenous control, the human β-actin gene (β-Act) was used. Reactions were run in duplicates or triplicates with 50 ng cDNA input material per detection well in a total reaction volume of 20 μl on an ABI PRISM 7900 HT Fast Real-Time PCR Sequence Detection System (ThermoFisher). Expression of *VENTX* was assayed by TaqMan^®^ quantitative real-time polymerase chain reaction (qRT-PCR) in the HEL erythroid leukemia as well as Kasumi-1, SKNO-1, HL60, NB4 and OCI-AML3 cell lines and in patient samples with AML M6 as well as polycythaemia vera and compared to patient samples with PML-RARα and sorted subpopulations of peripheral blood and BM from healthy donors. For VENTX the human TaqMan^®^ probe Hs00797729_s1 was used (ThermoFisher). Primer and probes for analyzing the expression of *VENTX* were selected specifically for that gene, in order to prevent amplification of known VENTX pseudogenes. The relative expression of each gene was determined by calculating fold change (2^-ΔCt^) to the housekeeping gene (β-Act).

### LM-PCR

For the linker-mediated PCR (LM-PCR), integrated Yellow and Green Fluorescent Protein (YFP and GFP) and flanking genomic sequences were amplified and then isolated using a modification of the bubble LM-PCR strategy [30]. Aliquots of the cell lysates from leukemic mouse bone marrow (BM) and spleen (SP) were digested with *PstI* (New England Biolabs Inc., Frankfurt a.M, Germany), and the fragments were ligated overnight at 16°C to a double-stranded bubble linker (5′CTCTCCCTTCTCGAATCGTAACCGTTCGTACGAGAATCGCTGTCCTCTCCTCCTGCA3′ and 5′GGAAGGAGAGGACGCTGTCTGTCGAAGGTAAGGAACGGACGAGAGAAGGGAGAG3′). Next, a first PCR (PCR-A) was performed on 10μl (one-tenth) of the ligation product using a linker specific Vectorette primer (5′CGAATCGTAACCGTTCGTACGAGAATCGCT3′) (ThermoFisher) and a GFP/YFP-specific primer (GFP/YFP-A: 5′ACTTCAAGATCCGCCACAAC3′) under the following conditions: 1 cycle of 94°C for 2 minutes, 30 cycles of 94°C for 30 seconds and 65°C for 1 minute, and 1 cycle of 72°C for 2 minutes. The bubble linker contains a 30-nucleotide non-homologous sequence in the middle region that prevents binding of the linker primer in the absence of minus strand generated by the LTR-specific primer. A 1μl-aliquot of the PCR-A reaction (one-fifteenth) was then used as a template for a second, nested PCR (PCR-B) using an internal GFP/YFP-specific primer (GFP/YFP-B: 5′ACATGGTCCTGCTGGAGTTC3′) and the same linker-specific Vectorette primer as was used in PCR-A, with the following conditions: 1 cycle of 94°C for 2 minutes, 35 cycles of 94°C for 60 seconds and 72°C for 1 minute, and 1 cycle of 72°C for 2 minutes. 100μl of the final PCR-B product were electrophoresed using a 2% agarose tris-acetate EDTA gel. Individual bands were excised and purified using the illustra GFX PCR DNA and Gel Band Purification Kit (GE Healthcare Life Sciences, Freiburg, Germany). The eluted samples were sequenced with the Vectorette primer by LGC genomics (Berlin, Germany). The sequences were then aligned using BLAST (www.ncbi.nlm.nih.gov/BLAST/) to identify the genomic location of the flanking sequences. Identified genomic loci were screened using the retroviral tagged cancer genes database (RTCGD).

### Retroviruses and plasmids

MSCV based retroviral vectors were used for overexpression of AML1-ETO (AE) and VENTX. The *VENTX* cDNA was provided by Paul Moretti (Institute of Medical and Veterinary Science, Adelaide, Australia) and subcloned into the retroviral pMSCV-IRES-YFP vector, as previously described [31]. The *AE* construct was subcloned into the GFP vector. As control, an empty vector (MSCV vector carrying only the IRES and GFP or YFP-cassette) was used (empty vector control).

### Transduction and BM transplantation

Stable packaging cell lines were generated for the different constructs and used for BM experiments as reported previously [21]. 5-FU BM was transduced with empty vector (control), VENTX, AE alone or with AE plus VENTX (AE/VENTX) in four, six, five and four independent experiments, respectively. Successfully transduced BM cells were transplanted unsorted into lethally irradiated (12 Gy) recipient mice. Transduction efficiency was on average 12.8% (2.2%-19.4%) or 0.7% (0.1%-1.2%) for VENTX alone and AE/VENTX double-transduced cells, respectively, 6.8-40.1% for the empty vector control and 26.1% (4.5%-36.9%) for AE. As donor mice > 12-week-old (C57Bl/6Ly-Pep3b x C3H/HeJ) F1 (PepC3) mice, as recipients > 12-week-old (C57Bl/6J x C3H/HeJ) F1 (B6C3) mice were used. The number of transplanted cells ranged from 4.5×10^5^ to 3.7×10^6^ cells per mouse for VENTX and from 4×10^4^ to 2×10^6^ cells per mouse for AE/VENTX (for AE 2.1×10^5^ – 2×10^6^ cells per mouse, for the empty vector control (ctrl.) 1.8×10^6^ – 3.3×10^6^ cells per mouse). For secondary transplantation between 8.7×10^4^ (plus once with 2.1×10^5^ helper cells) to 1×10^6^ unsorted cells from primary diseased mice were transplanted per mouse, for tertiary transplantation the number of transplanted cells per mouse ranged from 0.5 to 0.6×10^6^ unsorted BM cells from primary diseased animals.

CD34 positive human cord blood cells were retrovirally transduced with VENTX and the empty vector control as described previously [5]. After 48 hours successfully transduced cells were highly purified and subjected to RNASeq analysis.

### shRNA mediated VENTX knockdown

For stable shRNA mediated knockdown of endogenous *VENTX* genes, pLKO.1 based lentiviral vectors were used: pLKO.1 empty vector (SHC001), pLKO.1 scrambled (SHC002), pLKO.1-shVENTX_77 (TRCN0000015977, GenBank accession no. NM_014468.3), pLKO-1-shVENTX_73 (TRCN0000015973) (all obtained from Sigma-Aldrich, Munich, Germany). shVENTX_77 and shVENTX_73 oligonucleotides were cloned into the pGreenPuro vector (System Biosciences, California, USA). shRNA mediated knockdown in human cell lines was achieved following the experimental procedure as described previously [5].

For lentiviral transduction, Lenti-X™ 293T (Clontecth, #632180) cells were transiently transfected with CaCl_2_. Lenti-X™ 293T cells were cultured in a 10 cm dish to a confluency of 70%. Virus containing medium (VCM) was collected and filtered through a 0.45 μm filter. VCM was concentrated with an ultracentrifuge 1:100.

### Intracellular staining

For intracellular staining HEL cells were transduced in order to overexpress an HA-tagged construct of VENTX. Afterwards an shRNA mediated knockdown of VENTX was performed. 75,000 of these cells were spun with 450 rpm for 10 min onto microscope slides. Then, cells were fixed with 4% PFA for 10 min at RT, followed by a washing step in PBS. In order to permeabilize the cells, cells were treated with 0.1% Triton-X 100 for 5 min at RT. Afterwards cells were treated with pre-cooled pure Ethanol (cooled to -20°C) for 15 min at -20°C, before they were washed with PBS and blocked with 10% BSA for 1hr at RT. After blocking, cells were washed in PBS and incubated with the primary antibody anti-HA, rabbit (Abcam, 1:300 dilution) for 2 hrs at RT. Then, cells were washed with PBS and stained with the fluorescent secondary antibody goat-anti-rabbit-Alexa Fluor 594(1:1000 dilution) for 1 hr at RT in the dark. Then, cells were washed with PBS and nuclei were stained with DAPI (1:1000) for 3 min at RT. Cells were again washed with PBS and mounted with antifade fluorescent mounting medium and covered with a cover slip. Slides were analyzed with a fluorescent microscope.

### Colony forming cell (CFC) assay and cytospin

Hematopoietic colony forming cell (CFC) assays were performed using methylcellulose supplemented with murine cytokines (MethoCult GF M3434, Stem Cell Technologies, Cologne, Germany) as previously described [21]. 7 days after setting up the CFC, number and morphology of the colonies were assessed. For testing the clonogenic potential of leukemic BM from diseased mice 1-3×10^4^ cells per dish were plated for primary CFC and 500 to 5×10^3^ cells for re-plating assays. For cytomorphological analyses by cytospin 0.1×10^6^ cells were spun onto a microscope slide by a centrifugation step for 5 min at 350 rpm. The number of CFC for the first and second re-plating experiments was calculated for 1000 cells initially plated cells (CFC1), thereby accounting not only for the number of CFC per plated cell but also for the number of cells generated per CFC.

### Flow cytometric analysis

Cells were stained according to standard protocols. Cells were resuspended in PBS and treated with 1% ammonium chloride solution (10 min on ice). 1×10^5^ cells/FACS tube were resuspended in 100 μl of PBS and incubated on ice for 10 min with a purified rat anti-mouse CD16/32 antibody (BioLegend, Fell, Germany) to block non-specific antibody binding. After blocking, cells were stained for 20 min on ice. The following antibodies were used for flow cytometric analyses of murine bone marrow cells and permanently growing cells of a diseased mouse: Ter119 and CD36 labeled with APC (both BioLegend), B220 and Ter119 labeled with eFluor450 (both eBioscience, Frankfurt, Germany), Mac-1 labeled with qDot605 (BioLegend). Antibodies labeled with Alexa Fluor 700 were used against the epitope of c-kit (eBioscience), Gr-1 was labeled with APC-Cy7 (both BioLegend), Sca-1 with PE-Cy5.5 (Caltag) and CD71 with PE-Cy7 (Becton Dickinson and BioLegend, respectively). Cells were analyzed using a FACS FORTESSA LSR II (Becton Dickinson, Heidelberg, Germany).

### BrdU staining

BrdU staining was performed according to the manufacturer's instructions (BD Pharmingen BrdU Flow Kit, BrdU-APC). Briefly, 100,000 cells (HEL-SCR, HEL-shVENTX_73, HEL-shVENTX_77) were starved in RPMI supplemented with 1% FBS and 1% Pen/Strep for 16 hrs. Afterwards FBS was added in order to obtain a final concentration of 10% FBS and cells were incubated in that medium for another 24 hrs. BrdU treatment was performed for 30 min at 37°C.

### Annexin V staining

Annexin V apoptosis assay was performed according to the manufacturer's instructions (BD, APC Annexin V apoptosis Detection Kit).

### Bisulfite conversion and quantitative DNA methylation analysis (MassARRAY)

Bisulfite conversion and quantitative DNA methylation analysis (MassARRAY) was performed as described preciously [32]. Briefly, genomic DNA (gDNA) of sorted subpopulations from peripheral blood and cord blood from healthy donors was isolated according to the manufacturer's instructions using the column based DNeasy Blood & Tissue purification Kit (Quiagen, Hilden, Germany). Afterwards, gDNA samples were bisulfite-modified using the EZ DNA methylation kit (Zymo Research, Freiburg, Germany). The specific primer sequences used for PCR amplification are available upon request. The MassARRAY EpiTYPER Assay was performed at Sequenom Inc. (Hamburg, Germany) as described previously [33]. Amplicon 1 was located on chromosome 10:133237067-133237565, Amplicon 2 on chromosome 10: 133237570-133238054. The coordinates were based on genome version GRCh38/hg38 Dec 2013. After amplification, PCR products were transcribed *in vitro* before being cleaved by RNase A and subjected to matrix-assisted laser desorption/ionization time-of-flight mass spectrometry (MALDI-TOF MS). Methylation standards were used as controls and for normalizing data, while standards had 0%, 20%, 40%, 60%, 80%, and 100% of methylated genomic DNA.

### RNA-Seq methods

For cord blood experiments (VENTX, CD34^+^) sequencing libraries were prepared using Illumina TruSeq™ RNA Kit (Illumina, Inc.). All samples were run on the Illumina HiSeq2000 platform. The raw paired-end reads were adapter trimmed and quality filtered (phred score of > 20) using the cutadapt wrapper trim galore [34]. Filtered sequences were aligned to the human hg19 RefSeq using tophat and differential expression analyses were performed using Cufflinks with downstream analysis in R and Bioconductor [35–37].

### Gene set enrichment analysis

Gene Set Enrichment Analysis (GSEA) was performed using the GSEA tool from broad MIT [38], using the set of significantly differentially expressed genes from the RNA-Seq analysis generated from CB cells transduced with either VENTX or the empty vector control (n=3, respectively). GSEA focused on genes known to be involved in erythropoietic differentiation.

### Histopathology

For histological analyses, sections of selected organs were prepared and stained using standard protocols as previously described [4, 8].

### Statistical analyses

Data were evaluated using the *t* test for independent samples. Differences with *p* values less than 0.05 were considered to be statistically significant (* p≤0.05; ** p≤0.001; *** p≤0.0001; **** p<0.0001). Values mentioned are Mean ± SEM. PRISM GraphPad PRISM^®^ software, Prism 6 (Version 06.01) for Windows, Microsoft Excel 2010 was used to determine the correlation coefficients, Version 6.01 (La Jolla, California, USA) was used for the analysis and figures and FACSDiva 8.0 (Becton Dickinson) to analyze the FACS plots.

## SUPPLEMENTARY MATERIALS FIGURES AND TABLES










