# Supplementary material for: VENTX induces expansion of primitive erythroid cells and contributes to the development of acute myeloid leukemia in mice
Source: Oncotarget. 2016 Nov 24;7(52):86889–901. doi: 10.18632/oncotarget.13563 (PMC5349961; doi:10.18632/oncotarget.13563)
Supplement: Supplementary file 1 [file oncotarget-07-86889-s001.pdf]

# VENTX induces expansion of primitive erythroid cells and contributes to the development of acute myeloid leukemia in mice

## SUPPLEMENTARY FIGURES AND TABLES

A)

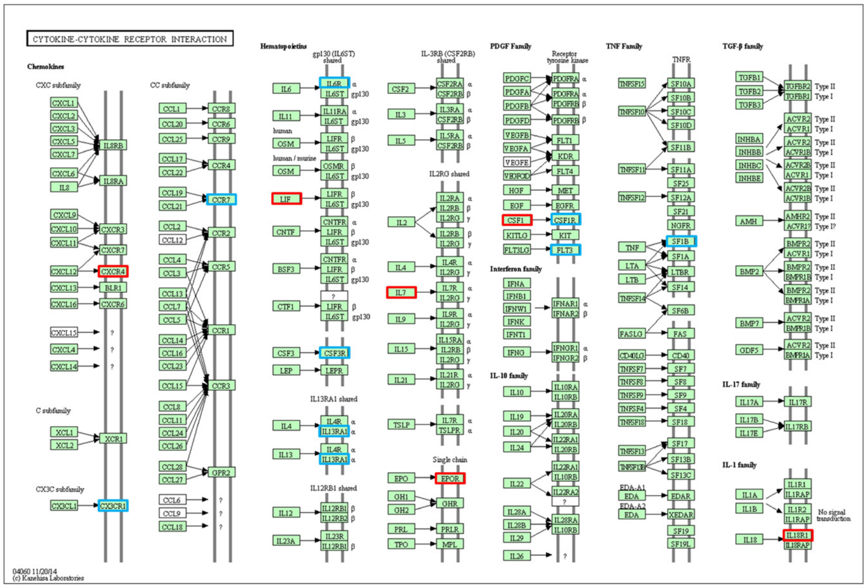


B)

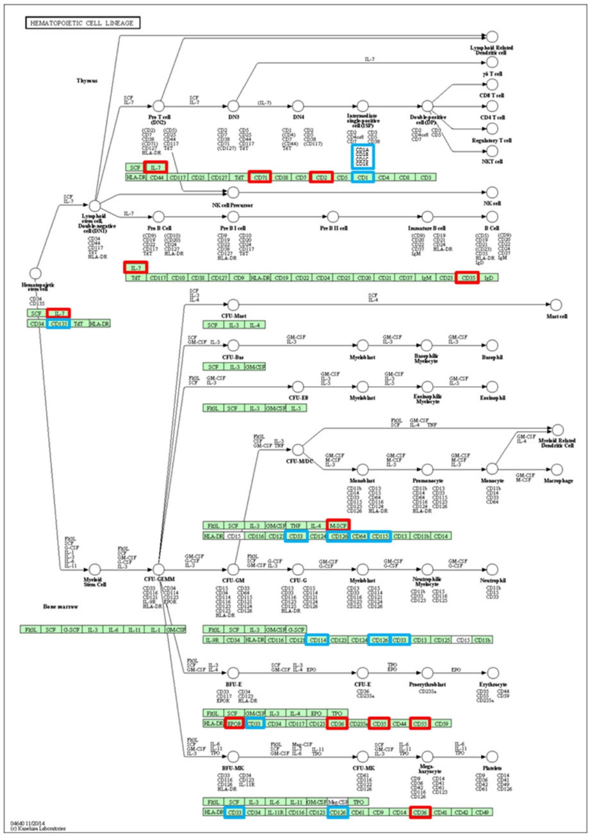


(*Continued*)

**C)**

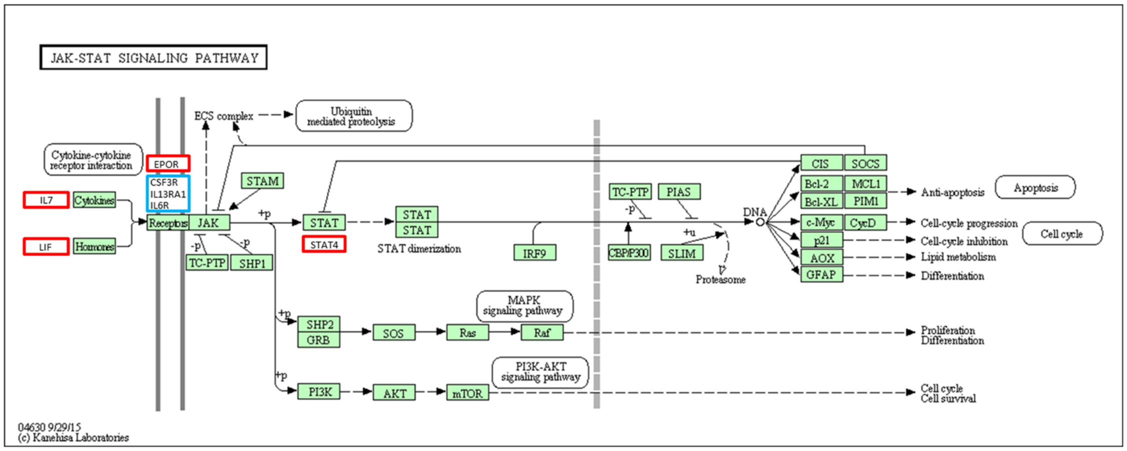


**D)**

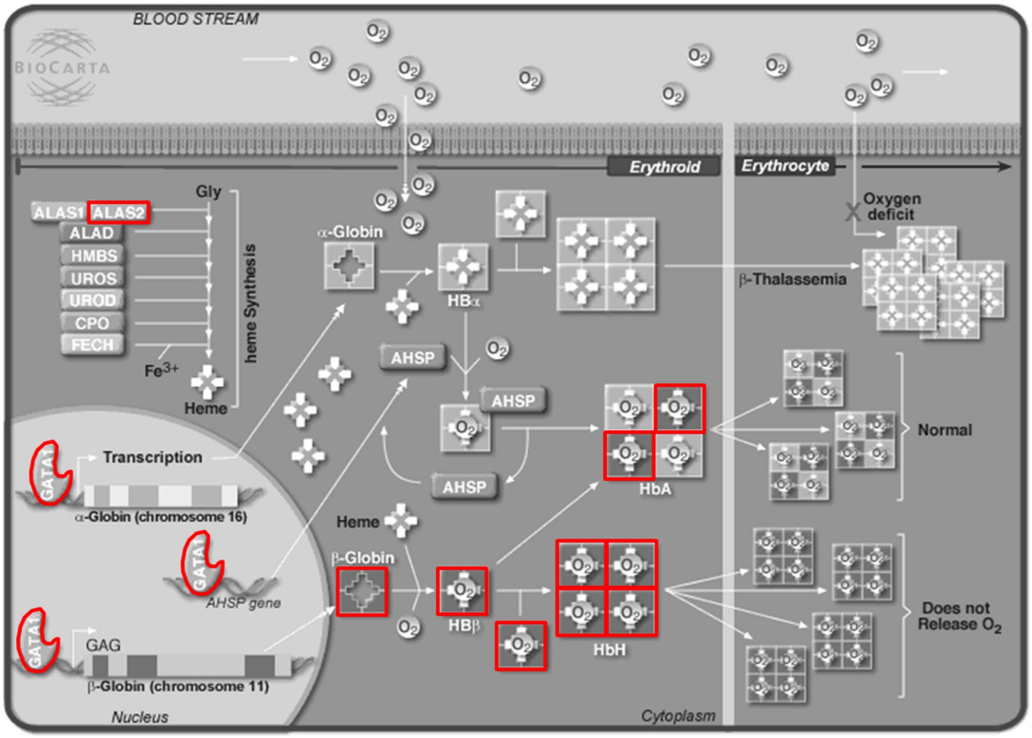


**Supplementary Figure S1:** Pathways according to A–C. DAVID and D. BIOCARTA.

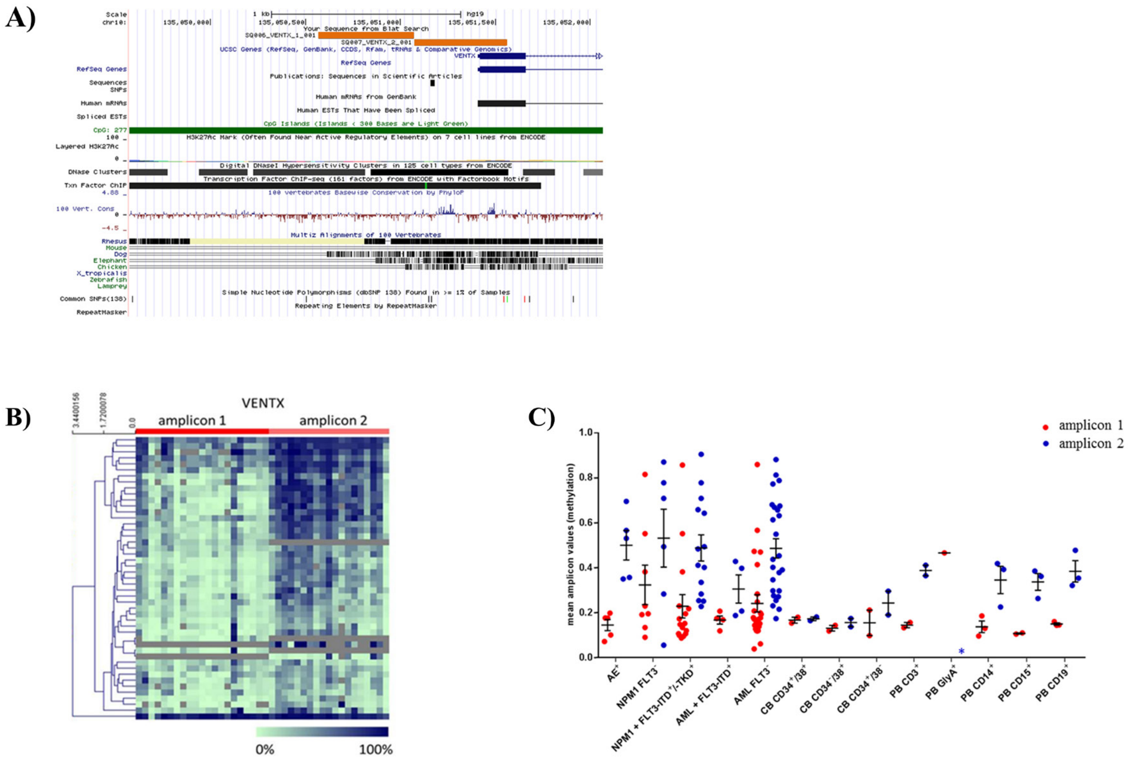


**Supplementary Figure S2: A.** VENTX promoter and coding sequence illustrated in the UCSC genome browser. **B.** Methylation levels shown as heatmap for *VENTX*. The heatmap displays single CpG units (columns). Methylation values range from 0% (light green) to 100% (dark blue). **C.** DNA methylation levels of *VENTX*. Average amplicon methylation is shown for individual patients for normal karyotype and t(8;21) positive AML. * In GlyA$^{+}$ sorted cells from peripheral blood amplicon 2 was not detected. The black bar represents the Mean ±SEM methylation. Each dot is representing one patient.

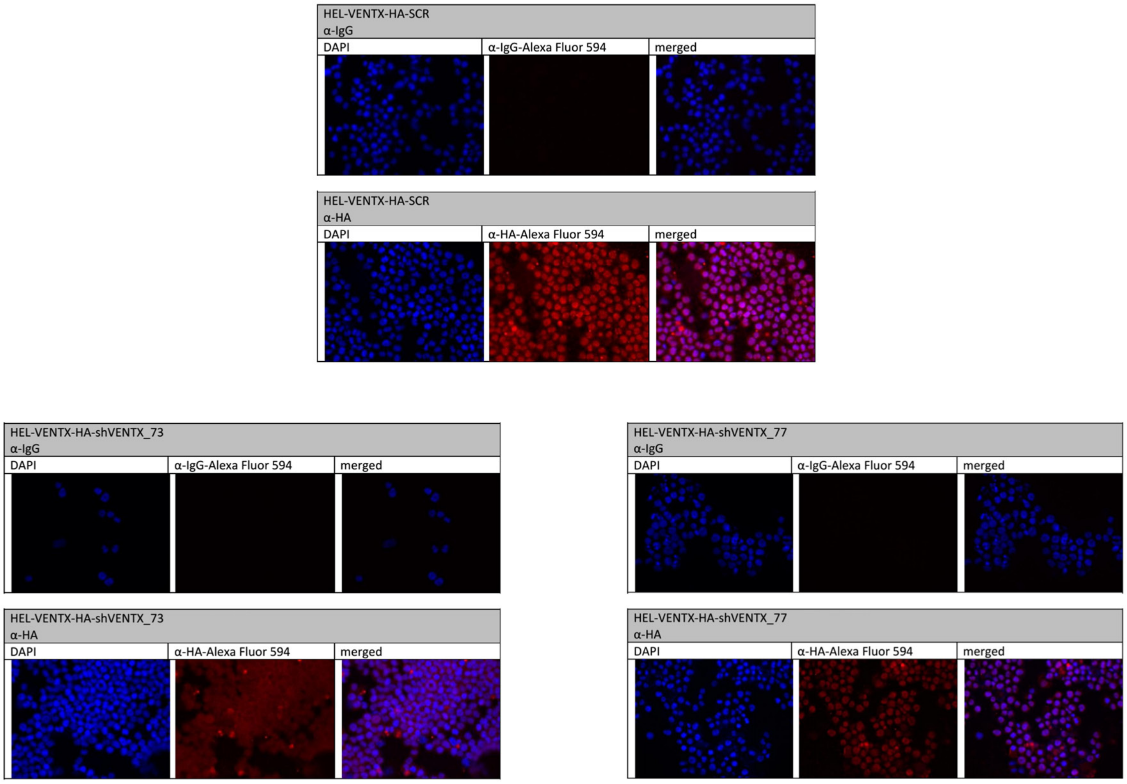


**Supplementary Figure S3:** Intracellular staining of HEL cells lentivirally transduced with a VENTX-HA tagged construct. Subsequently, knockdown was achieved using the scrambled control (SCR) versus shVENTX_73 as well as shVENTX_77 lentiviral constructs. Shown is the fluorescence after staining with an anti-HA versus an anti-IgG primary and an anti-rabbit-Alexa Fluor 594 secondary antibody (as described in material and methods).

A)

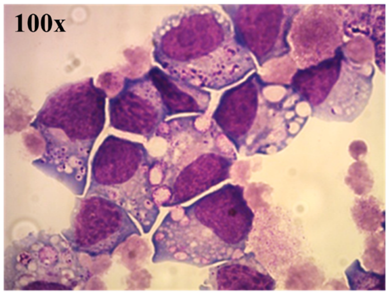


B)

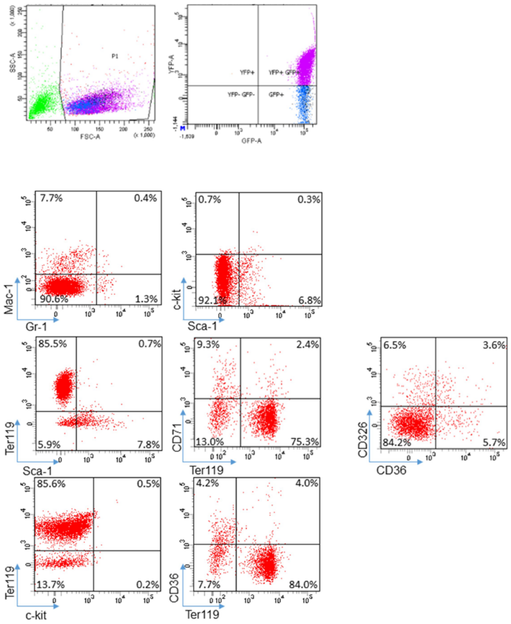


**Supplementary Figure S4: A.** Morphology and **B.** antigen profile of leukemic permanently growing cells established from a secondary AE/VENTX positive AML mouse.

**Supplementary Table S1: Genes differentially and significantly expressed in $CD34^+$ cord blood cells, comparing VENTX and empty vector control by RNA-Seq**

See Supplementary File 1

**Supplementary Table S2: Pathways according to DAVID and BIOCARTA**

See Supplementary File 2

**Supplementary Table S3: Differentially expressed genes overlapping with proteins regulated differentially in human erythropoiesis as shown in [1]**

See Supplementary File 3

**Supplementary Table S4: Characteristics of mice**

See Supplementary File 4

**Supplementary Table S5: Immunophenotype of mice**

| | Mouse # | Gr-1 | Mac-1 | Sca-1 | c-kit | Ter119 | CD71 | B220 |
|---|---|---|---|---|---|---|---|---|
| **VENTX 1°** | 62 | 44.3 | 43.7 | 46.8 | 49.5 | 2.7 | 21.3 | 2.2 |
| | 54 | 24.7 | 61.3 | 7.6 | 22.4 | 0.6 | 27.3 | 4.1 |
| | 61 | 75.8 | 0.5 | 7.5 | 19.2 | - | - | 2.2 |
| | 56 | 29 | 0 | 14 | 19.1 | 64.5 | - | 18.3 |
| **empty vector contorl 1°** | 43 | 56 | 59.4 | 7.39 | 5.2 | 22.71 | - | 6.41 |
| | 45 | 25.4 | 18.29 | 9.87 | 0.72 | 1.49 | - | 1.25 |
| | 38 | 74.5 | 65.3 | 2.9 | 1.8 | 40.9 | 30.4 | 0.4 |
| | 39 | 73.6 | 63.9 | 3.2 | 2.9 | 52.7 | 32.9 | 0.4 |
| **AE/VENTX 1°** | 9 | 34.7 | 67.1 | 32.4 | 24.6 | 1.9 | 39.3 | 7.4 |
| | 15 | 9.2 | 10.5 | 20.5 | 2.9 | 0.7 | 6.8 | 52.5 |
| | 6 | 37.4 | 33.2 | 17.6 | 5.2 | 9.7 | 10.4 | 48.3 |
| | 12 | 3 | 0 | 11.8 | 78.5 | 2.7 | 1.1 | 1.9 |
| | 17 | 52.3 | 51.4 | 27.2 | 8.5 | 3 | 12.4 | 20.3 |
| **AE 1°** | 21 | 5.4 | 1.4 | 83 | 0.7 | 5.2 | 49.9 | 2.7 |
| | 22 | 69.9 | 0 | 20.4 | 27.9 | - | - | 4.4 |
| | 35 | 42.58 | 45.22 | 15.82 | 4.15 | - | - | - |
| **AE/VENTX 2°** | 82 | 3.1 | 0.3 | 3.4 | 12.1 | 0.8 | - | 2.4 |
| | 80 | 73.3 | 3.7 | 7.1 | 18.8 | 1.7 | - | 2 |
| | 79 | 74.9 | 0.2 | 9.6 | 20.9 | 3.7 | - | 14.6 |
| | 76 | 92.5 | 20 | 8.4 | 12.7 | 18.7 | 71.9 | 2.6 |
| | 81 | 70.1 | 4.3 | 19.5 | 45.2 | 15.7 | 22.5 | 21.1 |
| | 75 | 1.1 | 16.9 | 1.8 | 59.6 | - | - | 0.2 |
| | 83 | 0.9 | 0 | 62.8 | 2.1 | - | - | 0.2 |
| | 77 | 73.9 | 91.6 | 3.1 | 10.3 | 1 | 47.7 | 6.4 |
| **VENTX 2°** | 86 | 0.3 | 0.1 | 0.4 | 0.5 | 95.2 | 0.7 | 0.1 |
| | 90 | 2.6 | 1.7 | 8.5 | 20.9 | 76.9 | 40 | 1.7 |
| | 91 | 42.2 | 4.4 | 12.8 | 96.6 | 91.3 | 79.3 | 4.1 |
| | 87 | 42.7 | 2.9 | 20.4 | 93.8 | 87 | 60.6 | 7.6 |
| | 89 | 48.1 | 7.3 | 20.9 | 94.2 | 86.6 | 49.9 | 4.2 |
| | 88 | 0 | 0.1 | 0.7 | 0.6 | 83.1 | 12.5 | 0.1 |

**Supplementary Table S6: Retroviral integration sites in leukemic BM**

| Mouse No. | LM-PCR Band No. | Chromosome | Genomic integration | RTCGD | Transcript Integration |
|---|---|---|---|---|---|
| 9 | 9_B1 | 7 | FMS-like tyrosine kinase 3 ligand (Flt3l) | - | FMS-like tyrosine kinase 3 ligand (Flt3l) |
| | | 12 | 5' side: sec1 family domain-containing protein 1, 3' side: cochlin precursor | - | |
| | 9_B2 | 11 | lymphocyte cytosolic protein 2 | - | |
| | 9_B3 | 11 | lymphocyte cytosolic protein 2 | - | |
| | 9_B4 | 11 | lymphocyte cytosolic protein 2 | - | |
| | 9_B5 | 11 | lymphocyte cytosolic protein 2 | - | |
| 15 | 15_B2 | 3 | ankyrin repeat domain 50 | - | |
| 41 | 41_B1 | 8 | DNA replication complex GINS protein PSF3 | - | |
| 43 | 43_B2 | 18 | 5' side: zinc finger protein 608, 3' side: GRAM domain-containing protein 3 | Present | |
| 22 | 22_B2 | 11 | 5' side: CMRF35-like molecule 2 precursor, 3' side: ras-related protein Rab-37 isoform 2 | - | |
| 48 | 48_B1 | 11 | 5' side: lymphocyte cytosolic protein 2, 3' side: forkhead box protein I1 | - | |
| 33 | 33_B2 | 4 | 5' side: protein FAM166B isoform 2, 3' side: dual specificity testis-specific protein kinase 1 | - | |
| 38 | 38_B2 | 2 | ral guanine nucleotide dissociation stimulator | - | |
| 50 SP | 50 SP_B1 | 14 | receptor-type tyrosine-protein phosphatase gamma precursor | - | |
| | 50 SP_B2 | 14 | receptor-type tyrosine-protein phosphatase gamma precursor | - | |
| | | 1 | striated muscle-specific serine/threonine-protein kinase | - | |
| | | 13 | spectrin alpha chain, erythrocytic 1 | - | |
| 50 BM | 50 BM_B1 | 6 | ras-related protein Rab-43 | - | |
| 49 SP | 49 SP_B1 | 18 | 5' side: phospholipid-transporting ATPase IC, 3' side: E3 ubiquitin-protein ligase NEDD4-like | - | E3 ubiquitin-protein ligase NEDD4-like transcript variant X6 |

## SUPPLEMENTARY REFERENCE

1. Gautier EF, Ducamp S, Leduc M, Salnot V, Guillonneau F, Dussiot M, Hale J, Giarratana MC, Raimbault A, Douay L, Lacombe C, Mohandas N, Verdier F, Zermati Y, Mayeux P. Comprehensive Proteomic Analysis of Human Erythropoiesis. Cell Reports. 2016; 161470-1484.
