# Supplementary material for: VENTX induces expansion of primitive erythroid cells and contributes to the development of acute myeloid leukemia in mice
Source: Oncotarget. 2016 Nov 24;7(52):86889–901. doi: 10.18632/oncotarget.13563 (PMC5349961; doi:10.18632/oncotarget.13563)
Supplement: Supplementary file 5 [file oncotarget-07-86889-s005.docx]

**Suppl. Table 4: Characteristics of mice**

| **Mouse no.** | **1°/2°/3°  Trans-plants** | **Retroviral construct** | **Days (d) post transplant** | **Spleen weight (mg)** | **WBC (PB) x10^6^/ml** | **RBC (PB) x10^9^/ml** | **PB (PLT) x10^6^/ml** | **% blasts BM** |
| --- | --- | --- | --- | --- | --- | --- | --- | --- |
| **1** | 1° | AE/VENTX | 385 | 119 | 1.8 | 5.75 | 1150 | 70 |
| **2** | 1° | AE/VENTX | 19 | 133 | NA | NA | NA | NA |
| **3** | 1° | AE/VENTX | 403 | 156 | 2.8 | 7.21 | 213 | 57 |
| **4** | 1° | AE/VENTX | 385 | 127 | 8.3 | 6.5 | 695 | 55 |
| **5** | 1° | AE/VENTX | 385 | 137 | 1.8 | 4.7 | 685 | 70 |
| **6** | 1° | AE/VENTX | 305 | 157 | 62.5 | 6.1 | 338 | 40 |
| **7** | 1° | AE/VENTX | 382 | 90 | 9.6 | 5.35 | 640 | 100 |
| **8** | 1° | AE/VENTX | 382 | 183 | 3.44 | 3.12 | 774 | 71 |
| **9** | 1° | AE/VENTX | 131 | 654 | 24* | 3.1* | 110* | 82 |
| **10** | 1° | AE/VENTX | 382 | 106 | 2.14 | 6.87 | 433 | 70 |
| **11** | 1° | AE/VENTX | 346 | 176 | 10.5* | 0.05* | 280* | 38 |
| **12** | 1° | AE/VENTX | 250 | 288 | 103.04 | 1.26 | 129 | 97 |
| **13** | 1° | AE/VENTX | 354 | 97 | 4.42 | 8.65 | 540 | 81 |
| **14** | 1° | AE/VENTX | 354 | 100 | 5 | 9.42 | 584 | 80 |
| **15** | 1° | AE/VENTX | 80 | 122 | 6.5 | 9.5 | 650 | 42 |
| **16** | 1° | AE/VENTX | 327 | 147 | 3.68 | 8.36 | 676 | 54 |
| **17** | 1° | AE/VENTX | 327 | 145 | 10 | 9.2 | 623 | 96 |
| **18** | 1° | AE/VENTX | 329 | 111 | 2.78 | 8.91 | 484 | 59 |
| **19** | 1° | AE/VENTX | 329 | 89 | 3.88 | 8.28 | 676 | 70 |
| **20** | 1° | AE/VENTX | 204 | 69 | 3.6 | 11.1 | 220 | 70 |
| **21** | 1° | AE | 21 | 34 | 2.72 | 0.74 | 30 | 0 |
| **22** | 1° | AE | 138 | 396 | 113.3 | 2 | 70 | 0 |
| **23** | 1° | AE | 96+ | NA | 12.4 | 7.3 | 420 | 0 |
| **24** | 1° | AE | 96+ | NA | 9.9 | 7.05 | 575 | 0 |
| **25** | 1° | AE | 96+ | NA | 8.3 | 5.75 | 370 | 0 |
| **26** | 1° | AE | 90 | 400 | 23* | 1.26* | n.d. | n.d. |
| **27** | 1° | AE | 365+ | NA | NA | NA | NA | NA |
| **28** | 1° | AE | 365+ | NA | NA | NA | NA | NA |
| **29** | 1° | AE | 178 | 270 | 5.2* | 2.0* | NA | NA |
| **30** | 1° | AE | 365+ | NA | NA | 7.0* | NA | NA |
| **31** | 1° | AE | 126 | 260 | 16* | 8.0* | NA. | 3 |
| **32** | 1° | AE | 262 | 223 | 13* | NA | NA | 0 |
| **33** | 1° | AE | 365+ | NA | NA | NA | NA | NA |
| **34** | 1° | AE | 365+ | NA | NA | NA | NA | NA |
| **35** | 1° | AE | 521 | NA | NA | NA | NA | NA |
| **36** | 1° | ctrl. | 327 | 124 | 3.42 | 8.65 | 600 | 0 |
| **37** | 1° | ctrl. | 327 | 194 | 6.14 | 9.45 | 539 | 0 |
| **38** | 1° | ctrl. | 329 | 195 | 5.5 | 8.12 | 409 | 0 |
| **39** | 1° | ctrl. | 329 | 370 | 4.26 | 4.62 | 901 | 0 |
| **40** | 1° | ctrl. | 96+ | NA | 13 | 8.35 | 845 | 0 |
| **41** | 1° | ctrl. | 96+ | NA | 13.4 | 7.6 | 765 | 0 |
| **42** | 1° | ctrl. | 96+ | NA | 14.2 | 8.7 | 785 | 0 |
| **43** | 1° | ctrl. | 90+ | NA | NA | NA | NA | 0 |
| **44** | 1° | ctrl. | 365 | NA | NA | NA | NA | NA |
| **45** | 1° | ctrl. | 119+ | NA | NA | NA | NA | NA |
| **46** | 1° | ctrl. | 272+ | NA | NA | NA | NA | NA |
| **47** | 1° | ctrl. | 365 | NA | NA | NA | NA | NA |
| **48** | 1° | ctrl. | 365 | NA | NA | NA | NA | NA |
| **49** | 1° | ctrl. | 365 | NA | NA | NA | NA | NA |
| **50** | 1° | ctrl. | 346 | NA | NA | NA | NA | NA |
| **51** | 1° | ctrl. | 263 | NA | NA | NA | NA | NA |
| **52** | 1° | ctrl. | 263 | NA | NA | NA | NA | NA |
| **53** | 1° | ctrl. | 263 | NA | NA | NA | NA | NA |
| **54** | 1° | VENTX | 189 | 128 | 8.2 | 10.4 | 910 | 29 |
| **55** | 1° | VENTX | 376 | 90 | 10.32 | 9.05 | 239 | 47 |
| **56** | 1° | VENTX | 376 | 89 | 11.46 | 9.15 | 436 | 47 |
| **57** | 1° | VENTX | 376 | 99 | 7.9 | 9.11 | 236 | 34 |
| **58** | 1° | VENTX | 354 | 237 | 3.04 | 6.64 | 200 | 77 |
| **59** | 1° | VENTX | 332 | 110 | 7.54 | 8.54 | 299 | 57 |
| **60** | 1° | VENTX | 332 | 122 | 6.08 | 9.13 | 722 | 64 |
| **61** | 1° | VENTX | 334 | 126 | 9.4 | 7.84 | 590 | NA |
| **62** | 1° | VENTX | 153 | 321 | 10.3 | 4.15 | 165 | 85 |
| **63** | 1° | VENTX | 215+ | NA | 24.3 | 9.3 | 1105 | NA |
| **64** | 1° | VENTX | 215+ | NA | 16.5 | 8.8 | 875 | NA |
| **65** | 1° | VENTX | 215+ | NA | 14.3 | 8.4 | 775 | NA |
| **66** | 1° | VENTX | 249 | NA | 9.6 | 7.8 | 605 | 60 |
| **67** | 1° | VENTX | 215+ | NA | 11.8 | 8.25 | 400 | NA |
| **68** | 1° | VENTX | 215+ | NA | 11.9 | 7.8 | 580 | NA |
| **69** | 1° | VENTX | 215+ | NA | 11.1 | 7.8 | 430 | NA |
| **70** | 1° | VENTX | 215+ | NA | 16.9 | 6.7 | 785 | NA |
| **71** | 1° | VENTX | 215+ | NA | 21 | 8.2 | 915 | NA |
| **72** | 2° | AE/VENTX | 343 | 157 | 2.96 | 7.88 | 575 | 51 |
| **73** | 2° | AE/VENTX | 343 | 142 | 5.28 | 10.64 | 269 | 59 |
| **74** | 2° | AE/VENTX | 343 | 392 | 4.7 | 6.69 | 582 | 65 |
| **75** | 2° | AE/VENTX | 35 | 169 | NA | NA | NA | 100 |
| **76** | 2° | AE/VENTX | 26 | 534 | 5.4 | 1.84 | 73 | 93 |
| **77** | 2° | AE/VENTX | 13 | 43 | 97.76 | 0.93 | 20 | 58 |
| **78** | 2° | AE/VENTX | 35 | 225 | 165.5 | 1.17 | 84 | 100 |
| **79** | 2° | AE/VENTX | 245 | 98 | 2.34 | 6.08 | 153 | 46 |
| **80** | 2° | AE/VENTX | 245 | 138 | 3.88 | 7.59 | 357 | 62 |
| **81** | 2° | AE/VENTX | 223 | 142 | 9.68 | 7.71 | 741 | 54 |
| **82** | 2° | AE/VENTX | 245 | 701 | 43.32 | 1.91 | 125 | 100 |
| **83** | 2° | AE/VENTX | 18 | 46 | 111.54 | 0.82 | 58 | NA |
| **84** | 2° | AE/VENTX | 35 | 258 | 2.3 | 3.45 | 100 | 90 |
| **85** | 2° | AE/VENTX | 28 | 72 | 133.34 | 0.53 | 86 | 95 |
| **86** | 2° | VENTX | 13 | 33 | 0.46 | 1.9 | 22 | 100 |
| **87** | 2° | VENTX | 16 | 72 | 22.86 | 3.38 | 143 | 100 |
| **88** | 2° | VENTX | 15 | 30 | 0.3 | 3.43 | 128 | 100 |
| **89** | 2° | VENTX | 16 | 108 | 0.74 | 2.83 | 121 | 85 |
| **90** | 2° | VENTX | 19 | 44 | 0.52 | 4.28 | 57 | 100 |
| **91** | 2° | VENTX | 16 | 127 | 0.64 | 4.57 | 208 | 83 |
| **92** | 2° | VENTX | 12 | 51 | 3.78 | 2.36 | 43 | 100 |
| **93** | 2° | VENTX | 13 | 34 | NA | NA | NA | 100 |
| **94** | 2° | VENTX | 14 | 175 | 8.1 | 5.42 | 393 | 100 |
| **95** | 2° | VENTX | 14 | 45 | 1.96 | 4.18 | 77 | 100 |
| **96** | 2° | VENTX | 14 | 42 | 1.34 | 4.31 | 75 | 100 |
| **97** | 2° | VENTX | 14 | 34 | 0 | 5.77 | 200 | 100 |
| **98** | 2° | VENTX | 11 | 28 | 142.14 | 3.98 | 62 | NA |
| **99** | 2° | VENTX | 148 | 61 | 128.68 | 8.85 | NA | NA |
| **100** | 2° | VENTX | 67 | 34 | NA | NA | NA | 46 |
| **101** | 2° | VENTX | 98 | 215 | 128.74 | 2.94 | 140 | NA |
| **102** | 2° | VENTX | 14 | 31 | 61.74 | 0.92 | 17 | NA |
| **103** | 2° | VENTX | 14 | 38 | 1.26 | 2.06 | 25 | NA |
| **104** | 2° | VENTX | 14 | 56 | NA | NA | NA | NA |
| **105** | 3° | AE/VENTX | 18 | 17 | 0.5 | 0.85 | 32 | 100 |
| **106** | 3° | AE/VENTX | 18 | NA | 0.22 | 1.02 | 36 | 100 |
| **107** | 3° | AE/VENTX | 14 | 40 | NA | NA | NA | NA |
| **108** | 3° | AE/VENTX | 18 | 24 | 0.28 | 1.68 | 25 | 100 |
| **109** | 3° | AE/VENTX | 18 | 26 | 0.56 | 0.63 | 22 | 100 |
| **110** | 3° | AE/VENTX | 13 | 27 | 0.36 | 1.74 | 473 | NA |
| **111** | 3° | AE/VENTX | 14 | NA | NA | NA | NA | NA |
| **112** | 3° | AE/VENTX | 14 | 32 | NA | 1.13 | 102 | NA |
| **113** | 3° | AE/VENTX | 14 | 21 | 6.48 | 1.82 | 34 | NA |
| **114** | 3° | VENTX | 18 | 25 | 1.06 | 1.07 | 53 | 95 |
| **115** | 3° | VENTX | 19 | 34 | 1.92 | 4.41 | 47 | 80 |
| **116** | 3° | VENTX | 19 | 115 | 1.14 | 4.05 | 62 | 70 |
| **117** | 3° | VENTX | 19 | 39 | 1.12 | 3.15 | 45 | 95 |

* values from lysed tissue, **Vegi et al., 2016, d+ mice still alive; NA = not available

AE = AML1-ETO, ctrl. = empty vector control
